# Complete genome sequence of *Flavobacterium* sp. strain CFS9, a potential fish probiotic isolated from the body surface of *Silurus asotus*

**DOI:** 10.1128/mra.00563-24

**Published:** 2024-11-06

**Authors:** Miho Kojima, Kaho Tobioka, Mika Okazaki, Kiyonobu Yokota, Dien Arista Anggorowati, Hajime Nakatani, Katsutoshi Hori, Yutaka Tamaru, Fumiyoshi Okazaki

**Affiliations:** 1 Department of Life Sciences, Graduate School of Bioresources, Mie University, Tsu, Japan; 2 Research Centre for Marine and Land Bioindustry, National Research and Innovation Agency (BRIN), North Lombok, West Nusa Tenggara, Indonesia; 3 Department of Biomolecular Engineering, Graduate School of Engineering, Nagoya University, Nagoya, Aichi, Japan; 4 Section of Soft and Functional Materials, Tohoku University Green Crosstech Research Center, Sendai, Japan; 5 Department of Applied Chemistry, Graduate School of Engineering, Tohoku University, Sendai, Japan; Montana State University, Bozeman, Montana, USA

**Keywords:** complete genome sequence, *Flavobacterium*, fish epidermal probiotics, *Edwardsiella ictaluri*

## Abstract

Here, we report the complete genome sequence of *Flavobacterium* sp. strain CFS9, a potential fish probiotic isolated from the body surface of *Silurus asotus*. The *de novo* assembly revealed a chromosome size of 5,370,016 bp with an estimated 4,374 open reading frames.

## ANNOUNCEMENT

*Flavobacterium* sp. strain CFS9 is a potential biocontrol agent against *Edwardsiella ictaluri*, a causative organism of enteric septicemia of catfish. The presented genomic information for strain CFS9 will facilitate the understanding of fish epidermal probiotics for aquaculture. We isolated the strain CFS9 from the body surface of cultured catfish *Silurus asotus* in 2018 (Table 1). Mucus samples were collected from seven *S*. *asotus* specimens and cultured on R2A agar plates at 25°C for 24–168 h to obtain the isolates. To screen isolates for their ability to inhibit the growth of *E. ictaluri*, they underwent a cross-streak test as previously described (1). Positive isolates were identified using molecular phylogenetic analysis of the 16S rRNA gene sequence. Consequently, the strain CFS9, which is classified within the genus *Flavobacterium* and exhibits the ability to inhibit the growth of *E. ictaluri*, was obtained.

**TABLE 1:** Genomic features of strain CFS9

Parameter	
Description of location	
Location	Shingu, Wakayama, Japan
Time	25 December 2018
Type	Body surface of *Silurus asotus* (cultured catfish)
Geographic coordinates	33°40′45.13N/135°57′45.66E
Sequencing statistics	
Number of raw reads	35,917
Mean length	6,929
Total bases	248,870,547
Number of Filtlong filtered reads	31,426
Genome statistics	
Assembly size (bp)	5,370,016
Number of contigs	1
GC content (%)	36.1
Genome coverage	46.3
Number of 5S rRNA	7
Number of 16S rRNA	7
Number of 23S rRNA	7
Number of tRNAs	70
Total number of CDS	4,374
Completeness (%)	99.7
Contamination (%)	0.93
Data accession	
BioProject	PRJDB18023
BioSample	SAMD00771247
GenBank accession no.	AP031573

The genomic DNA of the strain CFS9 was extracted using Genomic-tip 20/G (Qiagen), and the concentration of the DNA solution was measured using the QuantiFluor dsDNA System on a Quantus Fluorometer (Promega). Low-molecular-weight DNA was removed using the Short Read Eliminator (PacBio), and the DNA was fragmented to approximately 10–20 kbp using g-TUBE (Covaris). Fragmented DNA lengths were confirmed by measuring with a 5200 Fragment Analyzer (Agilent). The DNA library was prepared using the SMRTbell Express Template Prep Kit 2.0 according to the Procedure & Checklist instructions (Part Number 101-730-400 Ver. 06). Polymerase complexes were prepared by Binding Kit 2.2 (PacBio), and sequencing was performed using Sequel IIe (PacBio) by Bioengineering Lab. Co., Ltd. in 2023. SMRT Link (ver. 12.0.0.177059) was used to remove sequence adaptors, and consensus sequence reads with an average quality value of less than 20 per read were removed. Filtlong (version 0.2.1) was used to eliminate reads shorter than 1,000 bases and yielded 31,426 reads with an *N*_50_ value of 7,616 bp. *De novo* assembly was performed using Flye (ver. 2.9.1) (2, 3), and Bandage (ver. 0.8.1) (4) and CheckM (ver. 1.2.2) (5) were used for the quality assessment of the assembled genome. These results and other genomic information are shown in Table 1. Genome annotation was performed using the Prokka software (ver. 1.14.6) (6). Average Nucleotide Identity (ANI) was calculated using pyani (ver.0.2.9) (7). Digital DNA-DNA hybridization (dDDH) was calculated using GGDC (https://ggdc.dsmz.de/ggdc.php) (8, 9). When the strain CFS9 was compared with related species belonging to the genus *Flavobacterium*, ANI and dDDH were lower than the cut-off values of 95 and 70%, respectively. Therefore, strain CFS9 is considered to be a novel species within the genus *Flavobacterium*.

**Fig 1:**
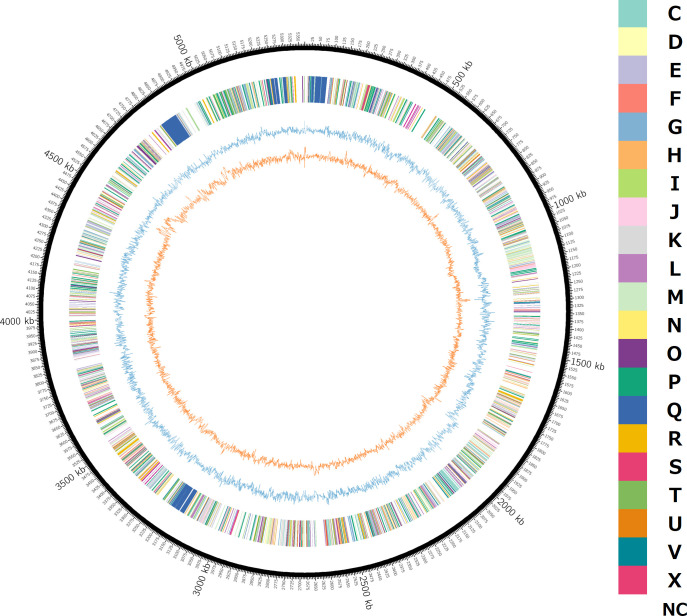
Circular representation of the genome of CFS9 strain. From outer circle to inner circle: color of inferred COG categories, GC skew (blue), GC contents (orange); NC in COG color code indicates no classified category.

The chromosome size of the strain CFS9 was 5,370,016 bp, and the contig was a single circularized genome. The genome coverage was 46.3×, and the GC content was 36.1%. The genome contains 4,374 coding sequences. Prokka predicted 91 RNA genes (70 tRNA genes and 21 rRNA genes). DFAST (https://dfast.ddbj.nig.ac.jp) (10, 11) predicted 4,305 protein-coding genes. COG categories were estimated using DFAST, and GC contents and GCskew were calculated using GCcalc (https://github.com/WenchaoLin/GCcalc). These results were visualized using Circos (ver. 0.69) (12) (Fig. 1).

## Data Availability

The whole-genome shotgun project for the strain CFS9 has been deposited in GenBank under accession number AP031573. The raw reads and raw sequencing data are available under BioProject accession number PRJDB18023 and BioSample accession number SAMD00771247.
